# Potential of *Panax ginseng* for bone health and osteoporosis management

**DOI:** 10.1016/j.chmed.2025.06.003

**Published:** 2025-06-27

**Authors:** Wenjie Fang, Kaisong Huang, Jinlian Hu

**Affiliations:** Department of Biomedical Engineering, City University of Hong Kong, Hong Kong 999077, China

**Keywords:** bone loss, ginsenosides, gut microbiota, herbal formulation, herb-drug interactions, medicine and food homology, osteoporosis, *Panax ginseng* C. A. Mey

## Abstract

*Panax ginseng* is a medicinal and food-homologous herb with a long-standing reputation as a tonic. Extensive research has explored its potential in improving osteoporosis, a condition characterized by progressive bone loss that increases the risk of fractures. In this review, PubMed, Web of Science, Google Scholar, China National Knowledge Infrastructure, and Chinese Medical Journal Database are searched with no lower time limit up to August 2024. This review offers a comprehensive overview of how *P. ginseng* and its formulations can enhance bone health and alleviate osteoporosis. It also explores the signaling pathways influenced by compounds derived from *P. ginseng* in bone turnover. *P. ginseng* shows promise in regulating sex hormones, exerting antioxidant and anti-inflammatory effects, and enhancing bone turnover. It suppresses MAPKs and NF-κB pathways to inhibit bone resorption, while activating pathways like Wnt/*β*-catenin, AMPK, PI3K/Akt, and BMP/Smad to promote bone formation. In addition, this review describes the global production and trade of ginseng, the metabolism of *P. ginseng* after oral ingestion, and network pharmacology studies. Given its multi-target mechanisms, *P. ginseng* shows promise in the clinical management of osteoporosis. At doses below 10 g with sustained use (several months to one year), it may serve as a viable daily supplement for bone health maintenance. Furthermore, its synergistic interactions with conventional anti-osteoporosis drugs (e.g., bisphosphonates) could enhance therapeutic efficacy, positioning it as a complementary adjunct in clinical treatment regimens.

## 1 Introduction

*Panax ginseng* C. A. Mey. is a medicinal and food-homologous herb. In East Asian, *Ginseng Radix* et *Rhizoma* has gained widespread recognition for its adaptogenic properties, which are believed to enhance the body's ability to cope with stress and promote overall well-being. In the United States, *P. ginseng* is classified as a dietary supplement under the Dietary Supplement Health and Education Act of 1994 (Miroddi, Mannucci, Mancari, Navarra, & Calapai, 2013). In 2023, the Chinese government also acknowledged *P. ginseng* as a raw material suitable for the production of healthcare foods and products. *P. ginseng* contains numerous active compounds that contribute to its medicinal effects. These effects encompass fatigue reduction, cognitive function improvement, immune function enhancement, diabetes management, support for sexual health, and alleviation of inflammation (Avsar et al., 2013, Kan et al., 2023, Kennedy and Scholey, 2003, Ling et al., 2024, Lu et al., 2023, Polan et al., 2004, Riaz et al., 2019, Tardy et al., 2021).

Living bone undergoes a continuous and dynamic remodeling process, where old bone minerals are lost and new ones are deposited. However, in osteoporosis, this delicate balance is disrupted, leading to a higher rate of bone loss compared to bone formation. Consequently, bones become thinner, more brittle, and prone to fractures (Compston, McClung, & Leslie, 2019). A systematic review of 103 334 579 people reports that the global prevalence of osteoporosis is 18.3 % (Xiao et al., 2022). A study on osteoporosis prevalence in China reveals that among adults aged 40 or older, osteoporosis rates were 5.0 % in men and 20.6 % in women. Notably, the prevalence of primary osteoporosis among older women is gradually elevated, with rates of 35.2 % for those aged 60–69, 44.1 % for those aged 70–79, and 53.9 % for those aged 80 and above. With the population aging, the incidence of osteoporosis is expected to rise further in China (Lyu et al., 2024, Wang et al., 2021). Numerous studies have indicated that *P. ginseng* holds promise in the treatment, protection, or prevention of osteoporosis (Liu et al., 2024). Research has shown that *P. ginseng* exhibits therapeutic effects on various aspects of osteoporosis, including estrogen deficiency, inflammation, oxidative damage, and imbalances in bone turnover (Ko, 2024, Park et al., 2017).

For methodology, we searched PubMed, Web of Science, Google Scholar, the China National Knowledge Infrastructure, and the Chinese Medical Journal Database. The search period ranged from the establishment of the database to August 2024. The following search terms were used: osteoporosis, bone loss, *P. ginseng*, ginseng, Renshen, Korean ginseng, red ginseng, white ginseng, ginsenoside, ginseng formulation, herbal formulation, drug-food interactions, and metabolism. This review aims to demonstrate the potential of *P. ginseng* for promoting bone health and managing osteoporosis.

## 2 Global ginseng production and trade

*P. ginseng* (Asian ginseng) and *P. quinquefolius* L. (American ginseng) are the primary raw materials used in the majority of ginseng products available on the market. As indicated in Table 1, China, South Korea, Canada, and the United States collectively dominate global ginseng production, accounting for over 99 % of the total. According to a 2022 report, these countries produced 58.18 %, 26.98 %, 13.18 %, and 1.49 % of the world's fresh ginseng, respectively (Baeg, 2022). Additionally, they collectively hold market shares of 48.64 %, 42.19 %, 8.10 %, and 0.92 % in the global ginseng production industry (Baeg, 2022). In comparison to a 2013 study conducted by Baeg, the total weight of ginseng production has increased from 80 080 to 86 223 tons in 2022. It is noteworthy that the price of Asian ginseng has experienced a significant surge, more than doubling its value since 2013. Conversely, the price of American ginseng has remained relatively stable. This price increase in Asian ginseng has contributed to the overall growth of the global ginseng production market, which has risen from $2 085 million to $5 900 million (Baeg & So, 2013).

**Table 1:** Variation of the global ginseng production market size based on the studies of (Baeg, 2022, Baeg and So, 2013).

Source	Ginseng production (ton) and ratio in the 2022 study	Ginseng production market size ($million) and ratio in the 2022 study	Ginseng production (ton) and ratio in the 2013 study	Ginseng production market size ($million) and ratio in the 2013 study
China	50 164 (58.18 %)	2 870 (48.64 %)	44 749 (55.87 %)	619 (29.69 %)
South Korea	23 265 (26.98 %)	2 489 (42.19 %)	27 480 (34.32 %)	1 140 (54.68 %)
Canada	11 367 (13.18 %)	478 (8.10 %)	6 486 (8.10 %)	269 (12.90 %)
United States	1 285 (1.49 %)	54 (0.92 %)	1 054 (1.32 %)	44 (2.11 %)
Others	142 (0.17 %)	9 (0.15 %)	311 (0.39 %)	13 (0.62 %)
Total	86 223 (100 %)	5 900 (100 %)	80 080 (100 %)	2 085 (100 %)

Ginseng is traded both domestically and internationally. In 2018, China ranked as the top importer of ginseng roots, with import values of $300.1 million. On the export side, China also takes the lead with $139 million, followed by Canada with $122 million, and South Korea with $79.5 million (Baeg, 2022). China and South Korea stand as the largest markets for ginseng root consumption, holding significant importance in traditional Chinese and Korean medicine practices. Moreover, ginseng has witnessed a surge in popularity as an ingredient incorporated into a wide range of food and beverage products. Examples include ginseng candy and ginseng chocolate, which have gained considerable traction among consumers (Chung, Lee, Rhee, & Lee, 2011).

## 3 Metabolism of *P. ginseng* after oral intake

*P. ginseng* is commonly consumed orally in various forms such as teas, capsules, or extracts. The gut microbiota plays a crucial role in the metabolism of ginsenosides. The process of intestinal metabolism involves the deglycosylation of ginsenosides at positions C3, C6, or C20, which is facilitated by the enzyme *β*-glucosidase (Kim et al., 2018). The gut microbiota produces therapeutic ginsenoside metabolites such as Rh_1_, Rh_2_, compound K, protopanaxadiol (PPD), and protopanaxatriol (PPT) (Yang et al., 2020). Moreover, intestinal metabolism significantly impacts the amount and type of ingredients absorbed. For example, compound K has been proven to improve fracture healing. After oral administration of *P. ginseng*, the amount of compound K absorbed into the bloodstream is correlated with the amount of compound K converted by the intestinal gut microbiota (Lee et al., 2009). Therefore, one reason for the different therapeutic effects of *P. ginseng* among individuals may be variations in gut bacteria. Furthermore, *P. ginseng* has been shown to have a positive effect on the gut microbiota. It acts as a modulator, selectively stimulating the growth and activity of beneficial bacteria in the gut (Chen et al., 2022). The correlation between *P. ginseng* and gut microbiota has become a focal point in current ginseng research.

The liver is also critical in the conversion of many drugs and xenobiotics into active metabolites. After 1 h intravenous of Rg_1_, the Rh_1_ and F_1_ metabolites appear in the blood, and the liver has the highest concentration of Rh_1_ and F_1_ (Feng, Wang, Hu, & Jiang, 2010). It suggests that the liver can transfer Rg_1_ to Rh_1_ and F_1_. In fact, liver enzymes can metabolize ginseng saponins. Cytochrome P450 3A4 (CYP3A4) functions as an isozyme in the metabolism of PPT-type ginsenosides. The order of oxygenation metabolism is as follows: Rf < Rg_2_ < Rh_1_ < PPT (Hao et al., 2010). However, there is insufficient information about the metabolism of ginsenosides in the liver or kidney. Liver metabolites (Rh_1_, Rg_2_) may improve bone turnover (Lee et al., 2023, Siddiqi et al., 2014a, Siddiqi et al., 2014b). These findings support the hypothesis that the liver metabolites of *P. ginseng* may enhance osteoporosis management.

## 4 Osteoporosis and its risk factors

### 4.1 Osteoporosis

**Fig. 1:**
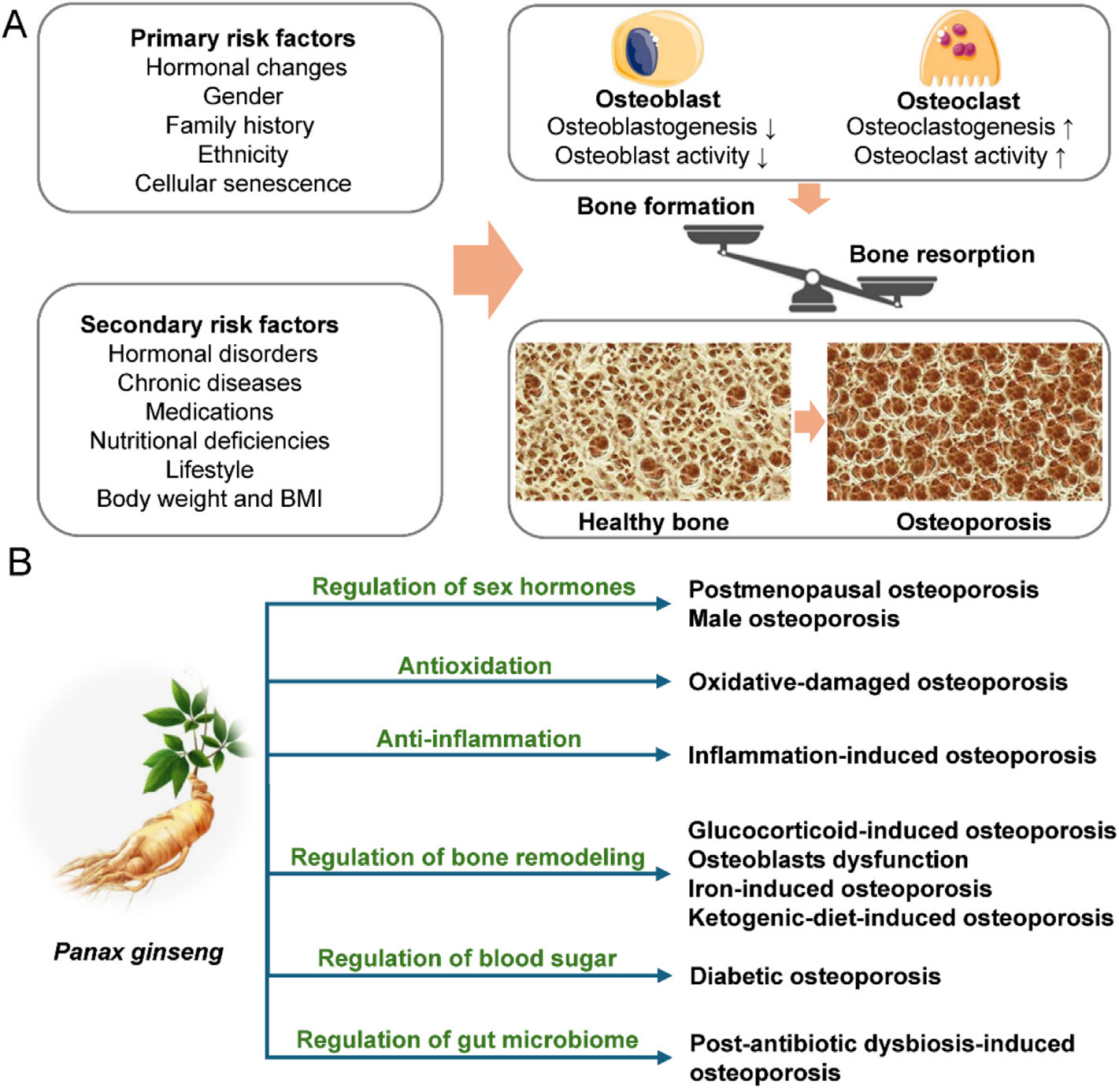
Primary and secondary risk factors cause abnormal bone turnover and contribute to the process of osteoporosis (A). Properties of *P. ginseng* have potential benefits in ameliorating various types of osteoporosis (B).

In clinical practice, the definitions of osteoporosis and osteopenia rely on the assessment of bone mineral density (BMD) using T-scores and Z-scores. These statistical measures provide valuable information about an individual's bone health by comparing their BMD to reference values (Kanis, 2008). Bone is a hard and rigid connective tissue composed of bone cells and bone matrix. One of its essential functions is to serve as a reservoir for minerals. In adults, the intricate process of bone remodeling involves osteoclast-mediated bone resorption, where bone minerals are removed, and the counterbalancing mechanism of bone formation, where new minerals are deposited by osteoblasts. However, as shown in Fig. 1A, when there is an excessive amount of resorption, it can lead to the gradual weakening and brittleness of bones over time, significantly elevating the risk of fractures (Compston et al., 2019).

### 4.2 Primary risk factors

The etiology of osteoporosis can be divided into two categories: primary and secondary risk factors, as indicated in Fig. 1A. Primary risk factors include aging, gender, genetics, and hormonal changes. These factors significantly contribute to the development of osteoporosis. In women, the decline in estrogen levels during menopause plays a crucial role. Estrogen has a protective effect on bone health, and its gradual loss leads to an accelerated rate of bone loss. Additionally, women generally have lower peak bone density compared to men. Peak bone density refers to the maximum amount of BMD an individual can achieve during their lifetime (Orwoll et al., 2001, Kalervo Väänänen and Härkönen, 1996). Similarly, in men, a reduction in testosterone levels can result in bone mineral loss (Mohamad, Soelaiman, & Chin, 2016).

Genetic factors also play a role in bone density, bone structure, and the body's ability to regulate bone remodeling. A positive family history of osteoporosis suggests the presence of shared genetic factors that contribute to lower bone density and an increased risk of fractures (Robitaille et al., 2008). Ethnicity is another factor influenced by genetics. The Caucasian and Asian populations have a higher prevalence of primary osteoporosis compared to other ethnic groups (Cauley, 2011). Furthermore, aging is associated with the accumulation of senescent cells. These cells undergo irreversible cell cycle arrest in response to stressors like DNA damage and telomere shortening. Senescent cells secrete a range of bioactive molecules and inflammatory factors known as the senescence-associated secretory phenotype (SASP). The SASP can stimulate pathological bone turnover, contributing to the development of osteoporosis (Föger-Samwald, Kerschan-Schindl, Butylina, & Pietschmann, 2022).

### 4.3 Secondary risk factors

Endocrine disorders that disrupt hormone levels, such as hyperthyroidism, hypogonadism, Cushing's syndrome, and hyperparathyroidism, can contribute to bone loss (Rosen, 1997). Additionally, individuals with chronic diseases, including rheumatoid arthritis, inflammatory bowel disease, chronic liver disease, chronic obstructive pulmonary disease, chronic kidney disease, multiple sclerosis, and acquired immunodeficiency syndrome, are at an elevated risk of developing secondary osteoporosis (Biver, 2022, Guañabens and Parés, 2018, Gupta et al., 2014, Llorente et al., 2020, Miller, 2014, Reinshagen, 2008).

Certain medications have also been associated with an increased risk of osteoporosis. Long-term use of glucocorticoids, commonly prescribed for conditions like rheumatoid arthritis, asthma, and lupus, can induce osteoporosis. Moreover, anticonvulsant drugs, cancer treatments (chemotherapy and hormonal therapies), and proton pump inhibitors used for acid reflux have been linked to an elevated risk of osteoporosis (Panday, Gona, & Humphrey, 2014). Unhealthy habits have also been found to have detrimental effects on bone health. Inadequate intake of calcium and vitamin D can lead to insufficient calcium absorption from the intestines (Heaney, 1993). Smoking and excessive alcohol consumption can reduce estrogen levels in women and impair the body's ability to absorb calcium and produce new bone tissue (Cheraghi et al., 2019, Weng et al., 2022). Furthermore, obesity is considered a significant trigger for fractures; low body weight is associated with a heightened risk of primary osteoporosis (Shapses & Riedt, 2006).

## 5 Therapeutic effects of *P. ginseng* on osteoporosis

### 5.1 Regulation of sex hormones

As indicated in Table 2, *P. ginseng* has been extensively studied for its potential therapeutic effects on various osteoporosis, particularly postmenopausal osteoporosis (PO). Women face a higher estimated lifetime risk of experiencing an osteoporotic fracture starting from the age of 50, with a ratio of one in two women being affected. In contrast, for men, the estimated lifetime risk is one in five (Eastell et al., 2016). The reduction in estrogen levels associated with menopause is a key factor in the development of PO, and *P. ginseng* has shown promise in managing menopause symptoms (Kim et al., 2013a, Kim et al., 2013b).

**Table 2:** Effects of *P. ginseng* on various osteoporosis.

Ingredients	Types of osteoporosis	Cell/animal/human & dose & *in vivo* delivery mode and duration	Therapeutic effects	References
*P. ginseng* extract	PO; age-related osteoporosis; PO with inflammation; GIO; post-antibiotic dysbiosis-induced osteoporosis	Postmenopausal women with osteopenia (1 and 3 g/d for one year, p.o.); male CD-1 mice (500 mg/(kg·d) for 4 w, p.o.); male Balb/C mice (500 mg/(kg·d) for 4 w, p.o.); male Wistar rats (300 mg/(kg·d) for 8 w, p.o.; 200 mg/(kg·d) for 6 w, p.o.); female Wistar rats (100 and 200 mg/(kg·d) for 2 months, p.o.; 100 to 500 mg/(kg·d) for 8 w, p.o.); male ICR mice (100 and 500 mg/(kg·d) for 4 w, p.o.); RAW264.7 cells (25 and 50 µg/mL); MC3T3-E1 cells (250 to 1 000 µg/mL)	Regulation of bone metabolism; hormonal effects; anti-inflammatory effects; anti-aging effects; regulation on the gut-bone axis	Avsar et al., 2013, Chargo et al., 2024, Ju et al., 2022, Jung et al., 2021, Kang et al., 2023, Kim et al., 2018, Kim et al., 2015, Kim et al., 2013b, Lee et al., 2015
Ginsenoside complex	PO	Female Wistar rats (10 to 30 mg/(kg·d) for 6 months, p.o.); MC3T3-E1 cells (1 and 10 μmol/L)	Regulation of bone metabolism	Gong et al., 2006
Rb_1_	Osteoblasts dysfunction; GIO	Female Sprague-Dawley (SD) rats (3 and 6 mg/(kg·d) for 12 w, i.m.); rat osteoblast (0.014 5 mg/mL)	Regulation of bone metabolism; anti-inflammatory effects	Zhang et al., 2022, Zhu et al., 2016
Rb_2_	Male osteoporosis; ketogenic-diet-induced osteoporosis; oxidative-damaged osteoporosis; GIO	Male C57BL/J mice (5 and 10 mg/(kg·d) for 8 w, p.o.); female BALB/c mice (4.6 and 18.5 μmol/(kg·d) for 12 w, p.o.); RAW264.7 cells (0.1 to 10 μmol/L); bone marrow macrophages (BMMs, 0.1 to 1 μmol/L); MC3T3-E1 cells (0.1 to 10 μmol/L); bone marrow-derived mesenchymal stem cells (BMSCs, 10 nmol/L)	Antioxidant properties; anti-inflammatory effects; anti-apoptotic effect on bone cells; regulation of bone metabolism	Gao et al., 2015, Huang et al., 2014, Ma et al., 2024; Liu et al., 2020
Rc	PO	Female C57Bl/6 mice (25 to 50 mg/(kg·d) for 3 months, p.o.); MC3T3-E1 cells (50 to 200 μmol/L)	Regulation of bone metabolism	Yang et al., 2022
Rg_1_	Diabetic osteoporosis; inflammation-induced osteoporosis	GK rats (110 mg/(kg·d) for 12 w, p.o.); rat osteoblasts (25 and 50 µg/mL); rat osteoprogenitors (164.8 μmol/L)	Promotion of angiogenesis and osteointegration coupling; anti-inflammatory effects	Chen et al., 2022, Lin et al., 2012
Rg_3_	PO, GIO; osteoblasts dysfunction	Female Wistar rats (2 mg/(kg·d) for 12 w, i.m.); male SD rats (20 and 64 mg/(kg·d) for 90 d, p.o.); female Sprague–Dawley rats (10 and 20 mg/(kg·d) for 5 w, p.o.; 20 mg/(kg·d) for 7 w, p.o.); rat osteoblasts (10 μmol/L); MC3T3-E1 cells (10 and 20 μmol/L)	Regulation of bone metabolism; antioxidant properties	Peng et al., 2020, Song et al., 2020, Zhang et al., 2016, Zhang et al., 2020

Ginseng harbors phytoestrogens, plant compounds capable of imitating estrogen's effects in the body. These phytoestrogens can interact with the estrogen receptor (ER) by either directly binding to it or indirectly triggering its activation. Rb_1_ activates both *α* and *β* estrogen receptors, and this activation is independent of directly binding to the ER (Cho, Park, Lee, Ahn, & Lee, 2004). Rg_1_ mediates the rapid extranuclear pathways at the plasma membrane (e.g. ER*α* and G protein-coupled receptor) to activate ER (Gao et al., 2019). Rh_1_ and Rh_2_ can act as ER ligands, exhibiting weak estrogenic activity (Lee et al., 2003, Peng et al., 2022). Additionally, *P. ginseng* extract at 0.5 mg/mL has been found to activate ER *in vitro* (Shim & Lee, 2012). *P. ginseng* at a daily dose of over 1 200 mg/(kg·d) for 7 d can upregulate the expression of ER in the reproductive organs of immature mice (Ding et al., 2015).

Androgens are also involved in maintaining bone mineral levels in men. Many clinical cases have reported that *P. ginseng* at doses of 800 mg to 3 000 mg can promote testosterone production (Wang et al., 2023). *P. ginseng* polysaccharides have been shown to upregulate mRNA expression levels of the androgen receptor (Park et al., 2017). These effects of *P. ginseng* may be associated with promoting the body's energy metabolism and increasing the abundance of probiotics, *Phascolarctobacterium* and *Roseburia* (Lin, Zhao, Liu, Liu, & Lin, 2021).

### 5.2 Antioxidation and anti-inflammation

Osteoporosis is associated with chronic inflammation and oxidative stress, both of which contribute to bone loss. *P. ginseng* possesses anti-inflammatory and antioxidant characteristics, which have the potential to mitigate inflammation and oxidative damage in bone tissues, potentially providing protection against osteoporosis (Li, Tian, Liu, Li, & Liu, 2023). *P. ginseng* extract, which was administered at a dose of 200 mg/(kg·d), has demonstrated a protective effect in an ovariectomy-induced osteoporosis model accompanied by magnesium-induced inflammation. Following a 60-d treatment period, rats exhibited restored BMD but still elevated levels of interleukin-1 (IL-1), IL-6, and tumor necrosis factor *α* (TNF-*α*) (Avsar et al., 2013). Furthermore, Rg_1_ has been identified as capable of reducing the production of pro-inflammatory cytokines by suppressing the cyclooxygenase-2-prostaglandin E2 pathway (Lin, Wu, He, Huang, & Lin, 2012). Additionally, Rb_2_ has been shown to diminish the generation of reactive oxygen species (ROS) induced by H_2_O_2_ through the c-Jun N-terminal kinase (JNK), macrophage stimulating 1 (Mst1) and Forkhead box O proteins (FoxOs) signaling pathways, thereby reducing oxidative damage and regulating the cytokines involved in bone resorption during osteogenesis (Huang et al., 2014).

### 5.3 Regulation of bone turnover

Glucocorticoid-induced osteoporosis (GIO) is characterized by the reduction of bone mass and an elevated risk of fractures resulting from long-term or high-dose glucocorticoid treatment. Glucocorticoids disrupt the delicate balance between bone formation and resorption, affecting bone metabolism (Compston, 2010). However, *P. ginseng* extracts in the range of 100−500 mg/(kg·d) offer potential protection against GIO and help prevent bone loss in mice (Chargo et al., 2024, Kim et al., 2015). Rg_3_ demonstrates the ability to mitigate GIO by reducing the number of osteoclasts and upregulating the expression of bone morphogenetic protein 2 (BMP-2) to promote osteoblastogenesis (Peng et al., 2020, Zhang et al., 2016). Similarly, Rb1 enhances osteoblast differentiation, thereby improving bone health (Zhang, Du, Yu, & Suo, 2022). Additionally, Rb_2_ effectively inhibits dexamethasone-induced apoptosis in bone marrow-derived mesenchymal stem cells (Gao et al., 2015).

Furthermore, elevated levels of aluminum in the body have been associated with an increased risk of osteoporosis. Aluminum can lead to decreased bone formation. However, Rb_1_ and Rg_3_ exhibit the potential to regulate oxidative stress and bone metabolism, thus mitigating osteoblast dysfunction (Song et al., 2020, Zhu et al., 2016).

## 6 Effects of *P. ginseng* extracts on bone turnover

### 6.1 Inhibition of bone resorption

**Fig. 2:**
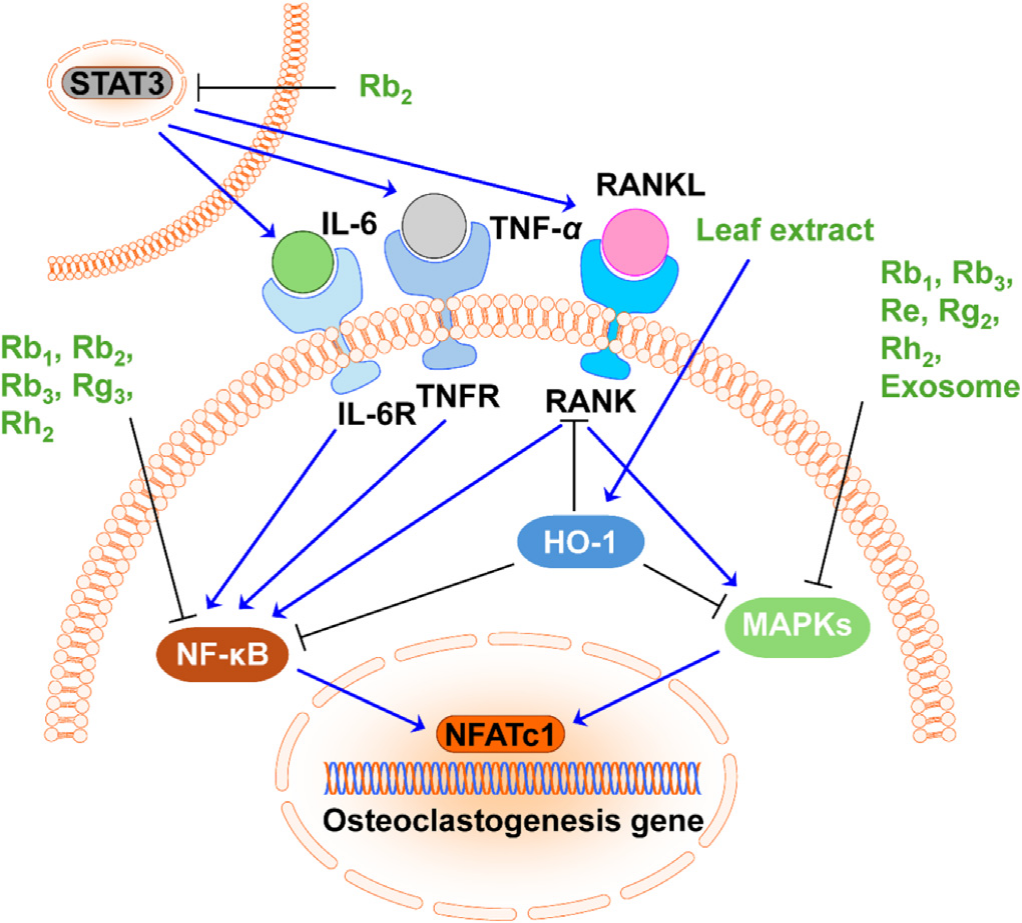
Effects of compounds derived from *P. ginseng* on bone resorption. Ginsenosides inhibit the STAT3, NF-κB, and MAPKs signaling pathways to suppress osteoclastogenesis.

Osteoporosis arises when there is an imbalance in bone remodeling. As listed in Table 3, numerous studies have shown that ginsenosides and other active compounds possess the ability to impede bone resorption and promote bone formation. Osteoclastogenesis refers to the process of osteoclast formation and maturation. Osteoclast progenitor cells undergo commitment to the osteoclast lineage as shown in Fig. 2. The receptor activator of nuclear factor kappa-B ligand (RANKL), produced by osteoblasts, stromal cells, and immune cells, plays a critical role in promoting osteoclast differentiation, fusion, and activation. It binds to its receptor, the receptor activator of nuclear factor kappa-B (RANK), on osteoclast precursor cells. Upon RANKL-RANK binding, multiple signaling pathways are triggered, such as nuclear factor kappa-light-chain-enhancer of activated B cells (NF-κB) and mitogen-activated protein kinases (MAPKs) (Wada, Nakashima, Hiroshi, & Penninger, 2006). NF-κB-mediated transcriptional activation of osteoclastogenic genes, particularly nuclear factor of activated T cells cytoplasmic 1 (NFATc1). NFATc1 acts as a master regulator of osteoclastogenesis and is essential for the expression of genes associated with osteoclast function. Several studies have demonstrated that Rb_1_, Rb_2_, Rb_3_, Re, Rg_2_, Rg_3_, Rh_2_, and *P. ginseng*-derived exosomes exert suppressive effects on RANKL-induced osteoclastogenesis by inhibiting NF-κB pathways and downregulating osteoclastogenic gene expression (Cheng et al., 2012, Cong et al., 2017, Lee et al., 2023, Liu et al., 2009, Park et al., 2016, Siddiqi et al., 2015, Sun et al., 2023).

**Table 3:** Effect of compounds derived from *P. ginseng* on bone turnover.

Compounds	Osteoclastogenesis	Osteoblastogenesis	Cell & dose	Pathways/receptors/cytokines	References
Rb_1_	↓		RAW264.7 cells (10 μmol/L)	RANKL/MAPK/NF-κB	Cheng et al., 2012
		↑	MC3T3-E1 cells (16 μmol/L)	Wnt/*β*-catenin	Zhou et al., 2018
Rb_2_	↓		RAW264.7 cells (10 μmol/L)	RANKL/NF-κB/STAT3	Cong et al., 2017
Rb_3_	↓		RAW264.7 cells (50 to 150 μmol/L); BMMs (50 to 150 μmol/L)	MAPK/NF-κB	Sun et al., 2023
Rc		↑	MC3T3-E1 cells (25 to 200 μmol/L)	Wnt/*β*-catenin	Yang et al., 2022
Rd		↑	MC3T3-E1 cells (40 μmol/L)	AMPK/BMP-2/Smad	Kim et al., 2012
Re	↓		BMMs (10 μmol/L)	RANKL/ MAPK	Park et al., 2016
		↑	MC3T3-E1 Cells (50 and 100 μmol/L)	Runx2	Kim et al., 2017
Rg_1_		↑	BMSCs (0.1 to 10 μg/mL; 50 μmol/L); MC3T3-E1 cells (50 μmol/L)	PI3K/AKt; BMP-2/Smad	Gu et al., 2016, Jiang et al., 2024
Rg_2_	↓		BMMs (40 μmol/L)	RANKL/MAPK	Lee et al., 2023
Rg_3_	↓		RAW246.7 cells (1 and 10 μmol/L)	RANKL/Cat-K/NF-κB	Siddiqi et al., 2015
Rg_5_/Rk_1_		↑	MC3T3-E1 cells (10 to 50 μmol/L)	BMP-2/Runx2	Siddiqi et al., 2014
Rh_1_		↑	MC3T3-E1 cells (10 to 100 μmol/L)	BMP-2/Runx2	Siddiqi et al., 2014a, Siddiqi et al., 2014b
Rh_2_	↓;		RAW246.7 cells (12 μmol/L); BMMs (12 μmol/L)	RANKL/NF-κB/MAPK	He et al., 2012, Liu et al., 2009
		↑	MC3T3-E1 cells (10 to 40 μmol/L)	PKD/AMPK;	Kim et al., 2011
Compound K		↑	BMSCs (10 μmol/L)	Wnt/*β*-catenin	Ding et al., 2022
Leaf extract	↓		RAW246.7 cells (1 mg/mL)	HO-1/ROS/HMGB1	Lee et al., 2024
Exosome	↓		BMMs (1 μg/mL)	RANKL/MAPK	Seo et al., 2023

Note: The ↓ indicates downregulation; The ↑ indicates upregulation; The blank indicates no record.

Moreover, MAPK signaling pathways also play important roles in regulating osteoclastogenesis. Three major MAPK pathways involved in osteoclastogenesis are the extracellular signal-regulated kinase (ERK), JNK, and p38 MAPK pathways. ERK and JNK, respectively, enhances the expression of c-Fos and c-Jun, which collaborate with NFATc1 to induce osteoclast-specific gene expression (Boyce et al., 2005). Furthermore, activation of p38 MAPK promotes osteoclast differentiation by regulating the expression and activation of NFATc1 and c-Fos. In addition, MAPK pathways are also involved in controlling osteoclast survival, proliferation, apoptosis, and the response to inflammatory cytokines such as IL-1, IL-6, and TNF-*α*. During the process of osteoclast attachment, JNK can influence actin cytoskeleton organization and actin ring formation (Lee, Seo, Choi, & Jeong, 2018). Rb_1_, Rb_3_, Re, Rg_2_, Rh_2_, and *P. ginseng*-derived exosomes are the reported active compounds for osteoporosis treatment, which can suppress MAPK signaling pathways and osteoclastogenesis (Cheng et al., 2012, Lee et al., 2023, Liu et al., 2009, Park et al., 2016, Sun et al., 2023).

As shown in Fig. 2, signal transducer and activator of transcription 3 (STAT3) can indirectly impact osteoclastogenesis via secreting cytokines to regulate NF-κB and MAPKs pathways. Activated STAT3 can lead to the production of cytokines and chemokines such as IL-1, TNF-*α* and RANK, which promote osteoclast differentiation and bone resorption. Rb_2_ can suppress the activation of STAT3 (Cong et al., 2017). Additionally, cathepsin K (Cat-K), a lysosomal cysteine protease, exhibits increased expression during osteoclast differentiation and is required for efficient fusion of precursor cells into multinucleated osteoclasts. Rg_3_ acts as an inhibitor against Cat-K expression (Siddiqi et al., 2015). Furthermore, elevated ROS levels have been shown to enhance NF-κB activation, leading to osteoclast differentiation. *P. ginseng* leaf extract has demonstrated the ability to enhance the expression of heme oxygenase-1 (HO-1), an inducible enzyme known for its antioxidant and cytoprotective properties. Through HO-1, *P. ginseng* leaf extracts can suppress ROS production and the secretion of high mobility group box 1 (HMGB1), a factor that can stimulate ROS production and osteoclastogenesis (Lee, Hur, Won, & Seo, 2024).

### 6.2 Promotion of bone formation

**Fig. 3:**
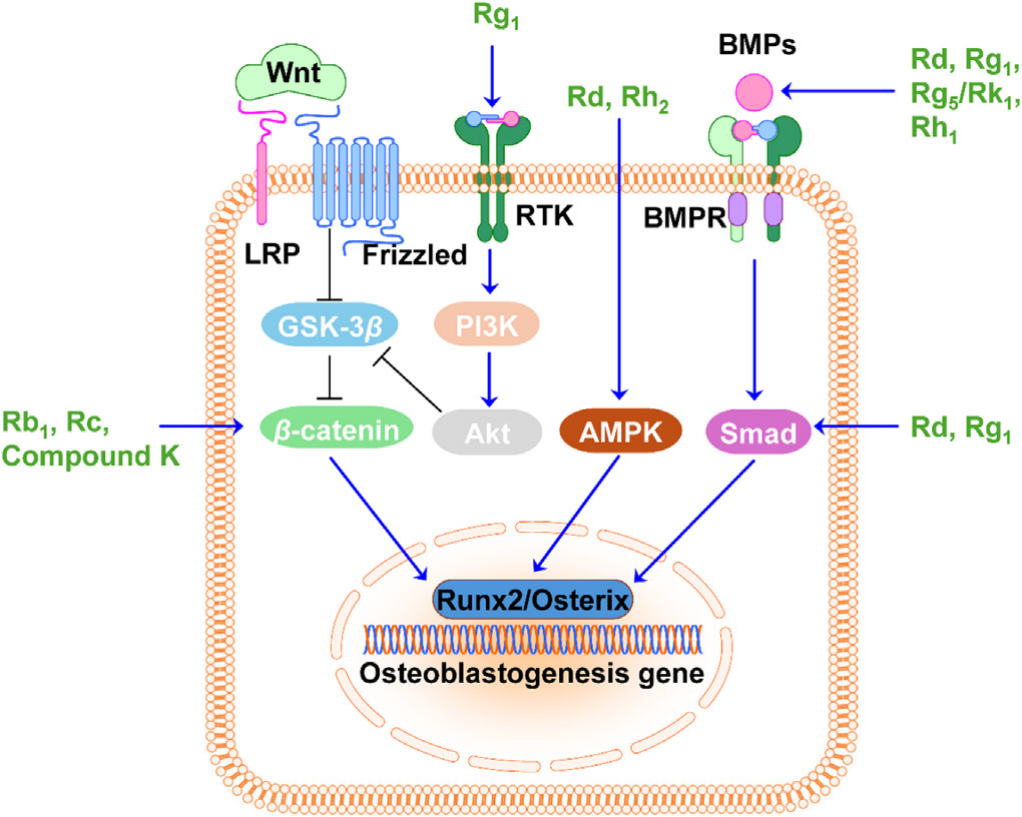
Effects of compounds derived from *P. ginseng* on bone formation. Ginsenosides activate the Wnt/*β*-catenin, PI3K/Akt, BMP/Smad, AMPK signaling pathways to promote osteoblastogenesis.

Bone formation is the process by which new bone tissue is produced. As illustrated in Fig. 3, the regulation of osteogenesis involves several signaling pathways, including the Wnt/*β*-catenin, bone morphogenetic proteins (BMPs), phosphoinositide 3-kinase/Akt (PI3K/Akt), protein kinase D (PKD), and AMP-activated protein kinase (AMPK). Activated Wnt prevents *β*-catenin phosphorylation and degradation through inhibiting glycogen synthase kinase 3*β* (GSK3*β*), leading to the cytoplasmic accumulation of *β*-catenin. Non-phosphorylated *β*-catenin enters the nucleus and activates the expression of Runt-related transcription factor 2 (Runx2). Runx2 acts as a master regulator of osteoblast differentiation, controlling the expression of genes involved in osteoblast function and matrix mineralization (Hartmann, 2006). Rb_1_, Rc, and compound K have been found to increase the expression of *β*-catenin and Runx2, thereby promoting osteogenesis (Ding et al., 2022, Yang et al., 2022, Zhou et al., 2018).

BMPs are a group of growth factors that bind to cell surface receptors. This binding leads to activation of the Smad pathway, where phosphorylated Smad proteins regulate the transcription of Runx2 and Osterix (Rahman, Akhtar, Jamil, Banik, & Asaduzzaman, 2015). Rd, Rg_1_, Rh_1_, and Rg_5_/Rk_1_ can enhance the expression of Runx2, and BMP receptor 1A. They can promote osteogenic differentiation to improve BMD and protect against fracture (Gu et al., 2016, Kim et al., 2012, Siddiqi et al., 2014a, Siddiqi et al., 2014b).

Furthermore, the PI3K/Akt pathway promotes osteoblast proliferation and differentiation through interactions with other signaling pathways. Activation of PI3K occurs in response to extracellular signals such as growth factors. Upon activation, PI3K phosphorylates to generate PIP3, which recruits Akt to the cell membrane. Akt is then phosphorylated and activated. Phosphorylated Akt inhibits GSK3*β*, leading to *β*-catenin signaling activation (Dong, Xu, Zhang, Yuan, & Tan, 2020). Rg_1_ has been shown to upregulate the expression of phosphorylated Akt (Jiang et al., 2024). In addition, Rh_2_ has been demonstrated to activate the PKD/AMPK pathway, thereby stimulating the expression of osteoblast-specific genes like Runx2 and osteocalcin (Kim et al., 2011b, Kim et al., 2011b).

## 7 Network pharmacology to explore the potential targets of *P. ginseng* on osteoporosis

Network pharmacology studies the interactions between drugs, targets and biological systems at a network level. Based on network pharmacology, Liu et al have explored the mechanism of *P. ginseng* in the treatment of osteoporosis. Twenty-two active ingredients from *P. ginseng* are screened from the traditional Chinese medicine systems pharmacology (TCMSP) database, including di-*iso*-octyl phthalate, stigmasterol, *β*-sitosterol, inermin, kaempferol, chrysanthemaxanthin, aposiopolamine, celabenzine, deoxyharringtonine, dianthramine, arachidonate, frutinone A, Rh_2_, Rh_4_, girinimbine, gomisin B, malkangunin, panaxadiol, suchilactone, alexandrine, Rg_5_, and fumarine. Gene ontology (GO) and Kyoto encyclopedia of genes and genomes (KEGG) enrichment analyses indicate that TNF, NF-κB, and the cancer-related pathway are the major pathways affected by *P. ginseng* (Liu, Zhang, Hu, Bai, & Guo, 2021).

Ginsenosides are the main therapeutic constituents of *P. ginseng*. A network pharmacology study has been conducted to uncover the potential targets of ginsenoside in osteoporosis. Rc, Rd, Rd_1_, Rg_3_, Rg_5_, Rh_2_, Rh_4_, 20-(*S*)-Rg_3_ are selected on the basis of oral bioavailability (≥30 %) and drug similarity (≥0.7). Then, by cross-validation of intersection and PPI network analysis, 14 potential key targets are obtained, which are IL-1*β*, matrix metalloproteinase-2 (MMP-2), TNF, interferon gamma (IFNG), Retinoid X receptor gamma (RXRG), RXRB, retinoic acid receptor beta (RARB), thyroid hormone receptor beta (THRB), capsase-1, 3, RARG, heme oxygenase 1 (HMOX1), NFKBIA and nuclear receptor coactivator (NCOA). Validated by enrichment analysis, IL-1*β*, TNF, IFNG and NFKBIA are the major target proteins. Molecular docking is then employed to predict the binding interactions of Rh2 with the four primary target proteins. Rh_2_ binds to several residues in NFKBIA, specifically phenylalanine 201 (F201), asparagine 205 (N205), lysine 208 (K208), aspartic acid 41 (D41), and serine 40 (S40). Additionally, it interacts with lysine 103 (K103) and methionine 148 (M148) in IL1B (Guo et al., 2022). The other research focuses on the important oral metabolite of *P. ginseng*, ginsenoside compound K. Five osteoclast differentiation-related pathways (PI3K-AKT, NF-κB, MAPK, Janus kinase/signal transducer and activator of transcription (JAK-STAT), and the calcium signaling pathway) are significantly enriched in KEGG analysis. Compound K has a poor permeability of cell membranes. c-Fms is the only membrane receptor protein enriched in the osteoclast differentiation pathway. According to molecular docking, compound K can bind to the cavity on the surface of c-Fms proteins with the lowest binding energy (−8.27 kcal/mol). Therefore, c-Fms-mediated MAPK and PIK3 signaling axis may be the potential mechanism for compound K in the treatment of osteoporosis (Zhang, Shen, Ma, & Fan, 2022).

## 8 *P. ginseng* for daily use and medical treatment of osteoporosis

*P. ginseng* is commonly consumed orally. The oral bioavailability of *P. ginseng* is generally low, with limited intestinal permeability. After a single administration, several ginsenosides, including R_1_, Ra_3_, Rb_1_, Rd, Re, Rg_1_, Rg_3_, Rh_1_, and Rh_2_, have been found to exhibit oral bioavailability of less than 10 % (Qi et al., 2011). However, multiple oral administrations can greatly enhance the absorption of ginsenosides compared to a single oral administration. A study involving 15 healthy Korean individuals demonstrated that the plasma concentration–time curve values of Rb_1_, Rb_2_, Rc, Rd, and compound K increased by 4.55, 6.73, 5.44, 4.49, and 0.85 times, respectively, following 15-d repeated administration (Choi et al., 2020). After absorption, the different ginsenosides are distributed throughout various tissues. For instance, Rg_1_ demonstrates extensive distribution in the heart, lungs, and kidneys. Re is primarily distributed in the heart and lungs. Rb_1_ predominantly distributes in the heart, while Rd is mainly found in the kidneys (Chen et al., 2018).

For daily use, David Kiefer and Traci Pantuso have recommended a daily dosage of 0.5 to 2 g of dried root and 200 mg of standardized extract of *P. ginseng* (Kiefer & Pantuso, 2003). The Ministry of Food and Drug Safety, Korea has indicated 0.5 to 5 g of powder or 3 to 80 mg of ginsenoside for daily intake (Jung et al., 2021). The 2020 edition of the *Chinese Pharmacopeia* has suggested 3 to 9 g/d of dry roots and 4 g/d of powder (Commission, 2020). A systematic review summarizes 82 clinical trials on *P. ginseng*. 26 studies used Korean red ginseng powder at doses ranging from 1 to 11.25 g/d, other studies used various forms of ginseng at daily doses ranging from 100 mg to 6 g. 15 studies report the following adverse events: diarrhea and gastrointestinal disorders, anxiety, sleep-related problems, epigastralgia, flu/cold, headache, contact urticarial reaction, itching, eye burning, improved motor efficiency, feelings of well-being and stimulation, increased appetite, skin eruptions, lighter hand and skin feeling ‘too tight’ (Coon & Ernst, 2002). In 1979, Ronald K. Siegel proposed the ginseng abuse syndrome (Siegel, 1979). Administration of *P. ginseng* in the long term or in an overdose has a certain risk of the occurrence of side effects. However, severe adverse effects are rare, and symptoms tend to subside upon discontinuation of ginseng use.

For medical treatment, Jung et al. conducted a 12-w, 90-subject, randomized, double-blind, placebo-controlled clinical trial to evaluate the effect of ginseng extracts (1 g/d or 3 g/d) on improving osteopathic and arthritis symptoms in women with osteopenia. The 3 g/d ginseng extract group has a significant improvement in serum markers, including osteocalcin and urinary deoxypyridinoline, and Western Ontario and McMaster Universities Osteoarthritis Index (WOMAC) index scores. No adverse effects were observed in this study (Jung et al., 2021). And no clinical study beyond one year. It is our contention that *P. ginseng* is not capable of aborting or reversing the process of osteoporosis in a prompt manner. *P. ginseng* is a medicinal and food-homologous herb. Taking less than 10 g of the herb daily for a period of several months to a year is considered safe. Should users experience any discomfort, we advise that they cease taking the herb.

## 9 Formulations containing *P. ginseng*

The Shang-Han-Lun is one of traditional Chinese medicine's oldest and most influential texts. A total of 13*P. ginseng*-containing prescriptions have been described in the Shang-Han Lun (Park et al., 2012). In traditional Chinese medicine, *P. ginseng* holds a revered status as one of the most esteemed herbs, recommended as a tonic, and consumed by the wealthy and noble classes. Ancient practitioners incorporated *P. ginseng* as a key ingredient in various classic formulations (Potenza, Montagnani, Santacroce, Charitos, & Bottalico, 2023). In emergency situations, high doses of red or white ginseng (processed *P. ginseng*) have been utilized as a medicinal intervention to revive comatose patients.

As indicated in Table 4, several studies have reported therapeutic, protective, or preventive effects of various *P. ginseng* formulations in improving bone remodeling and combating various osteoporosis (Al-Ghamdi and Alshaikh, 2021, Fan et al., 2023, Kang et al., 2020). Notably, formulations such as HER-S (300 mg/(kg·d) for 7 w) and Kang Shuai Lao Pian (450 mg/(kg·d) for 8 w) have demonstrated the potential to increase estrogen levels in models of estrogen deficiency associated with osteoporosis (Ekeuku et al., 2022, Kim et al., 2008). In addition, several formulations show therapeutic effects via anti-inflammatory and antioxidant properties. Bajitianwan (6 to 18 g/(kg·d) for 4 months) has been found to inhibit age-related osteoporosis induced by oxidative stress and alleviate memory impairments (Xu et al., 2024, Xu et al., 2020). The Qizhi Kebitong Formula (6 g/(kg·d) for 7 d) exhibits the ability to ameliorate osteoporosis complicated by diabetes through the inhibition of the PI3K/Akt/NF-κB pathway (Tian et al., 2022). Fufang Lurong Jiangu Capsule (1.8 g/(kg·d) for 8 d) offers protection to osteoblasts against oxidative damage induced by H_2_O_2_ (Jin et al., 2020).

**Table 4:** Studies on formulations containing *P. ginseng* for osteoporosis management.

Formulations	Types of osteoporosis	Cell/animal & dose & *In vivo* delivery mode and duration	Therapeutic effects	References
Red ginseng, *Angelicae Gigantis Radix*, *Phyllostachys Folium*, and soybean extracts (HER-S)	PO	Female SD rats (300 to 1 000 mg/(kg·d) for 7 w, p.o.); MC3T3E1 cells (0.1, and 1 μg/mL)	Increase in blood estrogen levels; prevention of bone loss	Kim et al., 2008
Red ginseng and *Lepidium sativum* L. seed extracts	PO	Female albino rats (100 mg/(kg·d) for 1 month, p.o.)	Increase in blood mineral levels (calcium, phosphorus, magnesium) and vitamin D; prevention of bone loss	Al-Ghamdi & Alshaikh, 2021
*Ginseng Radix* et *Rhizoma* and *Brassica oleracea* L. extracts	PO	Female C57BL/6 mice (500 mg/(kg·d) for 10 w, p.o.)	Inhibition of osteoclasts; prevention of bone loss	Kang et al., 2020
*Ginseng Radix* et *Rhizoma* and *Astragali Radix*	PO	Network pharmacology	Regulation of bone metabolism	Fan et al., 2023
Kang Shuai Lao Pian	PO	Female SD rats (0.15 to 0.45 g/(kg·d) for 8 w, p.o.)	Inhibition of bone resorption; prevention of bone loss	Ekeuku et al., 2022
Bajitianwan	Age-related osteoporosis; iron overload-induced osteoporosis	Male Wistar rats (6 and 12 g/(kg·d) for 4 months, p.o.); male C57/BL6 mice (9 and 18 g/(kg·d) for 12 w, p.o.); MC3T3-E1 cells (0.1 to 10 μg/mL)	Increase in levels of cytokines of bone formation; regulation of bone metabolism	Xu et al., 2024, Xu et al., 2020
Qizhi Kebitong formula	Diabetic osteoporosis	Male C57 BL/6 mice (1.5 to 6 g/(kg·d) for 1 w, p.o.)	Regulation of bone metabolism and anti-inflammation	Tian et al., 2022
Fufang Lurong Jiangu Capsule	Oxidative-damaged osteoporosis	C57BL/6 mice (500 mg/(kg·d) for 8 d, p.o.); BMSCs (50 to 200 μg/mL)	Antioxidant properties; promotion of bone formation	Jin et al., 2020

Some studies have explored the health benefits of combining *P. ginseng* with minerals and vitamins. Studies combining *P. ginseng* with vitamins and minerals (Gerovital®, G115®, Arginmax®) have shown that these formulations can improve immunity, vitality, cognition and female sexual function (Perazzo et al., 2010, Polan et al., 2004, Tardy et al., 2021). For osteoporosis, *P. ginseng*, calcium and vitamin D may have potential as a multicomponent fracture prevention supplement. Vitamin D is important for the absorption of calcium in the body. Daily calcium and vitamin D supplements are recommended to support BMD and prevent osteoporosis conditions like fractures (Aspray et al., 2014). The reason for adding *P. ginseng* is not only to benefit the bone tissue but also to benefit the body's vitality and mobility for fall prevention. In addition, vitamin C plays a key role in collagen synthesis, which forms the matrix of new bone. It is hypothesized that *P. ginseng* with vitamin C may promote osteogenesis.

As shown in Chapter 3, combining *P. ginseng* and probiotics can increase the effectiveness of both. Korean red ginseng can repair broad-spectrum antibiotic-induced intestinal microbiota dysbiosis, further alleviating bone loss by regulating the gut-bone axis (Kang et al., 2023). The herb-probiotic formulation will likely be an important product. Several studies have explored the interaction between *P. ginseng* and probiotics. For example, combining *P. ginseng* with the probiotic Lactobacillus exhibits antioxidant activity in a natural aging model and protects against liver diseases induced by a high-fat diet or alcohol (Bang et al., 2014, He et al., 2015, Kim et al., 2019). The potential of P. ginseng and probiotics in treating osteoporosis cannot be ignored.

## 10 Interactions of *P. ginseng* with anti-osteoporosis medications

*P. ginseng* has the potential to be incorporated into the guidelines for osteoporosis management. However, research concerning the interactions between *P. ginseng* and anti-osteoporosis medications is limited, particularly with rare clinical trials conducted thus far. Some medications can interact with each other, leading to harmful effects. A noteworthy clinical case has reported that an 82-year-old male patient experienced atorvastatin-induced liver injury only after concomitant ginseng intake. Clinicians suspect that ginseng's inhibition of cytochrome P450 enzymes (CYPs) may contribute to hepatotoxicity (Laube & Liu, 2019). CYPs are a superfamily of drug-metabolizing enzymes that play a crucial role in the metabolism of a wide variety of endogenous and exogenous compounds, including drugs, toxins, and environmental chemicals. The daily administration of *P. ginseng* extract powder in amounts less than 3 g has been found to have no significant impact on drug metabolizing enzymes and transporters (Choi & Song, 2019).

Moreover, *P. ginseng* has the potential to affect the therapeutic effects of medications. *P. ginseng* extract can enhance the cytotoxicity of chemotherapeutic agents (Chen et al., 2014). The intake of red ginseng and fermented red ginseng can effectively reduce cancer-related fatigue in patients undergoing platinum-based chemotherapy (Jiang et al., 2017). However, *P. ginseng* may also diminish the efficacy of specific drugs. *P. ginseng* can restore the levels of coagula factors suppressed by warfarin, as well as increase the activities of CYP 3A4 and 2C9 which metabolize warfarin (Dong et al., 2017). A clinical study reported that *P. quinquefolius* decreased the anticoagulant effects of warfarin (Yuan et al., 2004). However, another report suggested that co-administration of *P. ginseng* and warfarin did not affect the pharmacological effect of warfarin in ischemic stroke patients (Lee, Ahn, Ahn, Doo, & Lee, 2008).

The interaction between *P. ginseng* and anti-osteoporosis drugs should be studied, such as bisphosphonates, raloxifene, tibolone, denosumab, teriparatide, abaloparatide, and romosozumab. Tibolone is a steroid with weak estrogenic activity used in hormone replacement therapy for postmenopausal women (Kloosterboer, 2001). And raloxifene is a selective estrogen receptor modulator that has estrogenic activity in bone and liver (Prestwood et al., 2000). *P. ginseng* also has the ability to activate estrogen receptors and upregulate their expression. Therefore, combining *P. ginseng* with raloxifene and tibolone may potentially enhance the management of PO. In addition, bisphosphonates, the first-line treatment for osteoporosis, work by inhibiting bone resorption by interfering with intracellular enzyme functions (Compston, 2010). Denosumab is a monoclonal antibody that binds to and inhibits RANKL to inhibit osteoclastogenesis (Ferrari & Langdahl, 2023). Teriparatide and abaloparatide, which are two types of specific parathyroid hormone peptides, can stimulate bone formation more than bone resorption (Leder, 2017). *P. ginseng* is also considered a regulator of bone turnover. Co-administration can reduce the dosage of anti-osteoporosis drugs, therapy reduces the occurrence of side effects and may improve therapeutic effects.

## 11 Discussion

The potential of *P. ginseng* in promoting bone health and managing osteoporosis has been increasingly recognized through both traditional use and modern scientific research. This review has comprehensively explored the mechanisms by which *P. ginseng* and its bioactive compounds exert their effects on bone metabolism. The findings suggest that *P. ginseng* holds promise as a complementary approach to conventional osteoporosis treatments, particularly in addressing the multifactorial nature of the disease.

One of the key mechanisms through which *P. ginseng* exerts its beneficial effects on bone health is by modulating sex hormones, particularly estrogen and testosterone. Estrogen deficiency, a major contributor to PO, can be partially alleviated by the phytoestrogenic properties of ginsenosides, which interact with estrogen receptors and activate signaling pathways that promote bone formation (Shim & Lee, 2012). Similarly, *P. ginseng* has been shown to enhance testosterone production in men, which is crucial for maintaining BMD (Wang et al., 2023). The anti-inflammatory and antioxidant properties of *P. ginseng* further contribute to its therapeutic potential. Chronic inflammation and oxidative stress are known to exacerbate bone loss (Iantomasi et al., 2023). Ginsenosides have been shown to suppress pro-inflammatory cytokines and reduce oxidative damage, thereby protecting bone tissue from degradation.

Moreover, *P. ginseng* has the ability to influence key signaling pathways involved in bone metabolism. Rb_1_, Rb_2_, and Rg_3_ inhibit RANKL-induced NF-κB activation, thereby reducing osteoclastogenesis (Cheng et al., 2012, Cong et al., 2017, Siddiqi et al., 2015). Rg_2_ and Rh_2_ downregulate MAPK pathways, which are critical for osteoclast differentiation and survival (Lee et al., 2023, He et al., 2012). *P. ginseng* leaf extract enhances HO-1 expression, reducing ROS and HMGB1 secretion, which are implicated in osteoclast activation (Lee et al., 2024). On the other hand, Rc and compound K activate Wnt signaling, stabilizing *β*-catenin and promoting Runx2 expression (Ding et al., 2022, Yang et al., 2022). Rd and Rg_5_/Rk_1_ enhance BMP-2 expression and Smad phosphorylation, driving osteogenic differentiation (Kim et al., 2012, Siddiqi et al., 2014a, Siddiqi et al., 2014b). Rg_1_ activates PI3K/Akt, which inhibits GSK3*β* to amplify *β*-catenin signaling and osteoblast proliferation (Jiang et al., 2024). Rh_2_ stimulates AMPK and PKD pathways, upregulating osteoblast-specific genes like osteocalcin (Kim et al., 2011a, Kim et al., 2011b). By activating osteoblast differentiation and suppressing osteoclastogenesis, *P. ginseng* helps restore the balance between bone formation and resorption, which is often disrupted in osteoporosis.

The role of gut microbiota in the metabolism of *P. ginseng* also deserves attention. The gut-bone axis has emerged as a critical factor in bone health, and *P. ginseng*'s ability to modulate gut microbiota may contribute to its bone-protective effects (Zaiss, Jones, Schett, & Pacifici, 2019). By promoting the growth of beneficial bacteria and enhancing the production of bioactive metabolites such as compound K, *P. ginseng* may indirectly influence bone metabolism (Lee et al., 2009). This highlights the importance of considering the gut microbiome in future research on *P. ginseng* and osteoporosis.

Despite the promising findings, several limitations and gaps in the current research must be addressed. Most studies on *P. ginseng* and osteoporosis have been conducted at the cellular or animal level, with limited clinical evidence to support its efficacy in humans. Only one clinical trial has shown that oral administration of *P. ginseng* at a dose of 1 to 3 g/d for one year can improve some biomarkers such as osteocalcin, deoxypyridinoline and the WOMAC index (Jung et al., 2021). Larger, long-term clinical trials are needed to establish the safety, efficacy, and optimal dosage of *P. ginseng* for osteoporosis management.

Additionally, the potential interactions between *P. ginseng* and conventional anti-osteoporosis medications remain underexplored. While *P. ginseng* may enhance the therapeutic effects of drugs such as bisphosphonates and selective estrogen receptor modulators, there is also a risk of adverse interactions, particularly with medications metabolized by CYPs (Laube & Liu, 2019). Further research is needed to investigate these interactions and develop guidelines for the safe use of *P. ginseng* in combination with other treatments.

## 12 Conclusion

*P. ginseng* is a well-known medicinal and food-homologous herb. Currently, *P. ginseng* has shown therapeutic effects on 11 types of osteoporosis, including primary osteoporosis and secondary osteoporosis. Derived from *P. ginseng*, 15 compounds have been proven to improve bone turnover. In addition, 8 herbal formulations containing *P. ginseng* have been developed for osteoporosis. They provide comprehensive evidence supporting that *P. ginseng* has the potential to manage osteoporosis and enhance bone health. However, most studies are still in the cellular or animal levels. It lacks strong evidence of clinical effectiveness. In this review, we provide a hypothesis of daily doses and duration of ginseng administration. We hope more professionals can join in this career to integrate *P. ginseng* into daily osteoporosis management.

## CRediT authorship contribution statement

**Wenjie Fang:** Data curation, Formal analysis, Visualization, Writing – original draft. **Kaisong Huang:** Writing – review & editing. **Jinlian Hu:** Conceptualization, Supervision, Data curation, Project administration, Validation, Writing – original draft, Writing – review & editing.

## Declaration of competing interest

The authors declare that they have no known competing financial interests or personal relationships that could have appeared to influence the work reported in this paper.
